# Absorbed Dose–Response Relationship in Patients with Gastroenteropancreatic Neuroendocrine Tumors Treated with [^177^Lu]Lu-DOTATATE: One Step Closer to Personalized Medicine

**DOI:** 10.2967/jnumed.123.267023

**Published:** 2024-06

**Authors:** Kévin Hebert, Lore Santoro, Maeva Monnier, Florence Castan, Ikrame Berkane, Eric Assénat, Cyril Fersing, Pauline Gélibert, Jean-Pierre Pouget, Manuel Bardiès, Pierre-Olivier Kotzki, Emmanuel Deshayes

**Affiliations:** 1 Department of Nuclear Medicine, Institut du Cancer de Montpellier, Université de Montpellier, Montpellier, France;; 2 Institut de Recherche en Cancérologie de Montpellier, INSERM U1194, Université de Montpellier, Montpellier, France;; 3 Biometry Unit, Institut du Cancer de Montpellier, Université de Montpellier, Montpellier, France;; 4 Department of Medical Oncology, CHU de Montpellier, Université de Montpellier, Montpellier, France;; 5 IBMM, Université de Montpellier, CNRS, ENSCM, Montpellier, France; and; 6 Centre Hospitalier Métropole Savoie, Chambéry, France

**Keywords:** [^177^Lu]Lu-DOTATATE, neuroendocrine tumors, absorbed dose–response relationship

## Abstract

[^177^Lu]Lu-DOTATATE has been approved for progressive and inoperable gastroenteropancreatic neuroendocrine tumors (GEP-NETs) that overexpress somatostatin receptors. The absorbed doses by limiting organs and tumors can be quantified by serial postinfusion scintigraphy measurements of the γ-emissions from ^177^Lu. The objective of this work was to explore how postinfusion [^177^Lu]Lu-DOTATATE dosimetry could influence clinical management by predicting treatment efficacy (tumor shrinkage and survival) and toxicity. **Methods:** Patients with GEP-NETs treated with [^177^Lu]Lu-DOTATATE between 2016 and 2022 and who underwent dosimetry were included. Absorbed doses were calculated for healthy organs (liver, kidneys, bone marrow, and spleen) and tumors using PLANET Dose and the local energy deposition method based on serial posttreatment SPECT/CT. Up to 5 lesions per site were selected and measured on images collected at baseline and 3 mo after treatment end (measurement masked to the somatostatin receptor imaging uptake). For toxicity assessment, laboratory parameters were regularly monitored. Clinical data, including time to death or progression, were collected from the patients’ health records. Correlations between absorbed doses by organs and toxicity and between absorbed doses by lesions and tumor volume variation were studied using regression models. **Results:** In total, 35 dosimetric studies were performed in patients with mostly grade 2 (77%) tumors and metastases in liver (89%), lymph nodes (77%), and bone (34%), and 146 lesions were analyzed: 1–9 lesions per patient, mostly liver metastases (65%) and lymph nodes (25%). The median total absorbed dose by tumors was 94.4 Gy. The absorbed doses by tumors significantly decreased between cycles. The absorbed dose by tumors was significantly associated with tumor volume variation (*P* < 0.001) 3 mo after treatment end, and it was a significant prognostic factor for survival. Toxicity analysis showed a correlation between the decrease of hematologic parameters such as lymphocytes or platelet concentrations and the absorbed doses by the spleen or bone marrow. The mean absorbed dose by the kidneys was not correlated with nephrotoxicity during the studied period. **Conclusion:** In patients treated with [^177^Lu]Lu-DOTATATE for GEP-NETs, tumor and healthy organ dosimetry can predict survival and toxicities, thus influencing clinical management.

## Visual Abstract

**Figure:**
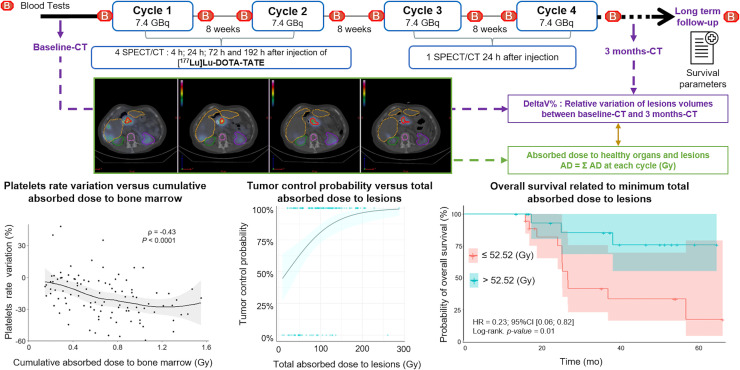

Well-differentiated neuroendocrine tumors (NETs) are a heterogeneous tumor type derived from the diffuse endocrine system. The primary sites are mostly the gastroenteropancreatic system and lungs. The World Health Organization proposed a prognostic classification of NETs in 3 grades based on the histologic features of proliferation (1). NETs usually overexpress somatostatin receptors (SSTRs), especially in grade 1 and grade 2 tumors. Since the 1990s, peptide receptor radionuclide therapy (PRRT) has been developed using radiolabeled somatostatin analogs, initially with ^111^In (2) and ^90^Y and more recently with ^177^Lu. The NETTER-1 prospective randomized multicenter phase III trial showed an improvement in progression-free survival and health-related quality of life in patients with progressive midgut grade 1 or grade 2 NETs treated with [^177^Lu]Lu-DOTATATE compared with patients receiving a high dose of long-acting octreotide (3*,*^4^). These results and a previous cohort study (5) led to the approval by regulatory authorities of [^177^Lu]Lu-DOTATATE (Lutathera; AAA/Novartis) for the treatment of progressive or inoperable, well-differentiated gastroenteropancreatic NETs (GEP-NETs). This treatment has been reimbursed in Europe since 2017 and in the United States since 2018. The recommended regimen with [^177^Lu]Lu-DOTATATE, derived from the NETTER-1 trial, consists of 4 intravenous injections of fixed activities (7.4 GBq) separated by an interval of 8 wk. This regimen is well tolerated by most patients. However, it does not take into account the full potential of [^177^Lu]Lu compounds that can be imaged after injection to calculate the absorbed dose (AD) by organs and lesions because of coemission of γ-particles (6). The AD is a physical parameter that is expressed in grays and reflects the amount of energy in joules released by ionizing radiation and absorbed per unit mass of tissue (in kilograms). For a given radionuclide and a given cell type, the damage to cells progressively increases with the AD. It has been shown that when [^177^Lu]Lu-based PRRT ([^177^Lu]Lu-PRRT) is given with a fixed regimen, the ADs by organs and tumors are heterogeneous among individuals (7*,*^8^). This variability is directly correlated with the drug biodistribution and its residence time in the organs of interest. In [^177^Lu]Lu-PRRT, the relationships between ADs by healthy organs and biologic effects (i.e., toxicities) have been studied mostly using kidney dosimetry and kidney function assessment. Different methods have been proposed to calculate the ADs by kidneys after [^177^Lu]Lu-PRRT, but none found a correlation between ADs and acute and middle–to–long-term kidney impairment (9*,*^10^). Concerning hematologic toxicities, some retrospective cohort studies found significant correlations between the AD of bone marrow (11*,*^12^) or the spleen (13) and the decrease of blood count parameters (i.e., platelets, white blood cells, hemoglobin). In addition, the relationship between the AD by tumors and efficacy has been little studied. A few studies found a positive correlation between the AD by the small intestine or pancreatic NETs and tumor shrinkage (14*,*^15^), a tumor volume decrease based on SSTR molecular imaging (16), and recently that the AD by NETs can predict patient survival (17). The aim of this study was to explore how postinfusion [^177^Lu]Lu-DOTATATE dosimetry in patients with GEP-NETs treated with a fixed regimen could influence the clinical management by predicting treatment efficacy (tumor shrinkage and survival) and toxicity.

## MATERIALS AND METHODS

### Patients and Treatments

Patients who were treated with [^177^Lu]Lu-DOTATATE (Lutathera; AAA/Novartis) between 2016 and 2022 for progressive GEP-NETs overexpressing SSTRs and who underwent imaging for dosimetric purposes after each [^177^Lu]Lu-DOTATATE cycle were included in this retrospective single-center study. They received an intravenous injection of a fixed activity of 7.4 GBq of [^177^Lu]Lu-DOTATATE every 8 wk (≤4 injection cycles in total). To avoid nephrotoxicity, an intravenous injection of amino acid solution (1 L, 25 g of arginine, and 25 g of lysine) was delivered to patients over 4 h, starting 30 min before [^177^Lu]Lu-DOTATATE infusion. Clinical and biologic data were extracted from the patients’ health records. The study was approved by the institutional ethics review board (ICM-ART 2023/03).

### Dosimetry Workflow

The calibration steps of the SPECT/CT Discovery NM/CT 670 system (GE Healthcare) are detailed in the supplemental materials (available at http://jnm.snmjournals.org). For cycles 1 and 2, SPECT/CT images with at least 1 field of view that included the kidneys and liver were planned at 4, 24, 72, and 192 h after injection of [^177^Lu]Lu-DOTATATE. For cycles 3 and 4, a single-time-point SPECT/CT acquisition was performed 24 h after injection, aligning with the patient’s release timing. [^177^Lu]Lu-DOTATATE scintigraphy acquisitions were performed and reconstructed according to a previously described protocol (18). Briefly, a medium-energy general-purpose collimator was used. For 60 projections at 45 s each, the energy window was set at 208 keV ± 20%, with a 10% scatter window centered at 177 keV. Attenuation, scatter, and recovery resolution corrections were applied. The first CT scan (4 h after injection) was acquired with 120 kV, automatic milliampere regulation, a slice thickness of 5 mm, a rotation time of 0.8 s, a pitch of 1.375, and a pixel matrix of 512 × 512. All other CT scans were acquired with parameters inducing lower irradiation (rotation time, 0.6 s; 80 mA fixed). Dosimetry was performed using the European Conformity–marked PLANET Dose software, version 3.1.1.83 (DOSIsoft SA). An automatic and rigid registration based on the first CT image, taken as a reference, was performed for all SPECT/CT images of the same cycle. The volumes of interest of healthy organs (spleen, kidneys, liver, and trabecular sections of lumbar vertebrae 2–4, representing bone marrow) were manually segmented on the reference CT image and then propagated and adjusted to the subsequent SPECT/CT images. For lesions, the volumes of interest were initially delineated on baseline, pretreatment, and contrast-enhanced CT (BL-CT) images and then drawn on scintigraphy images on the basis of an isocontour that corresponded to the volume defined on the BL-CT image. For the first 2 cycles, time-absorbed dose-rate curves were produced and fitted using a monoexponential model. ADs were calculated using the local energy deposition method with density correction. To simplify the procedure, for cycles 3 and 4, ADs were calculated with the following equation,

$$\text{AD}\;\text{cycle 3 or 4}=\text{AD}\;\text{cycle}\;2\times \frac{\text{counts}\;24\;\text{h}\;\text{cycle 3 or 4}}{\text{counts}\;24\;\text{h}\;\text{cycle}\;2}\times \frac{\text{V}\;24\;\text{h}\;\text{cycle}\;2}{\text{V}\;24\;\text{h}\;\text{cycle 3 or 4}},$$

where AD cycle 2 is the AD calculated after cycle 2 (with 4 time points), counts 24 h is the number of counts in structures segmented on the SPECT/CT images acquired 24 h after [^177^Lu]Lu-DOTATATE injection, and V 24 h is the volume of the related structure. A partial-volume–effect correction was performed by applying to AD a recovery coefficient based on the calibration studies (supplemental materials).

### Choice of Lesions of Interest

Target lesions were selected on the BL-CT images, masked to their SSTR expression status, and manually contoured to produce a volume of interest. They were categorized by the following sites: liver, lymph nodes, mesenteric mass, pancreas, and peritoneum. A maximum of 5 lesions per organ or site was allowed per patient (including the lesions with the highest and smallest volume in each organ). Bone lesions were excluded because of the difficulty in assessing the response to treatment. Lesions smaller than 2 cm^3^ were excluded to minimize the partial-volume effect (2 cm^3^ corresponds to a recovery factor of 0.5; Supplemental Fig. 1). The lesion volume was reassessed on the contrast-enhanced CT images performed 3 mo after the last [^177^Lu]Lu-DOTATATE injection (M3-CT). Variations in the lesion volume between BL-CT and M3-CT were defined as

$$\text{DeltaV\%}=100\times \frac{\text{V}\left[\text{M}3\text{-CT}\right]-\text{V}\left[\text{BL-CT}\right]}{\text{V}[\text{BL-CT}]},$$

where DeltaV% is the variation of lesion volume and V[M3-CT] and V[BL-CT] are the lesion volumes of M3-CT and BL-CT, respectively.

### Collected Data and Studied Parameters

Laboratory parameters were collected from the patients’ health records before the first cycle and around day 15 after each cycle, including hemoglobin, white blood cell count, platelet count, creatinine concentration, and glomerular filtration rate, which was estimated using the creatinine serum levels and the Chronic Kidney Disease Epidemiology Collaboration formula. Treatment efficacy was assessed using RECIST 1.1 and by comparing BL-CT and M3-CT images. Toxicity was graded according to the Common Terminology Criteria for Adverse Events (version 4.03). Cumulative AD (CumAD) is defined as the sum of the AD calculated after each cycle for the same PRRT course and the sum used to correlate with toxicity (Supplemental Fig. 2). To define the dosimetric indices for lesions, the following variables were studied: the mean of the total AD of all lesions in each patient and the lesions with the highest (Max) and the lowest (Min) total AD among all lesions in each patient.

### Statistical Analysis

Qualitative variables were described using the number of observations and the frequency of each modality. Percentages were calculated excluding missing data. Quantitative data were described using the medians, the minimum, and the maximum or the means and the SD. Variables of interest were dichotomized using the medians. The statistical analyses are fully described in the supplemental materials. All analyses were done with SAS version 9.4 software (SAS Products & Solutions) and R version 4.0.3 software (The R Project for Statistical Computing).

## RESULTS

In total, 34 patients met the inclusion criteria. Among them, 2 patients were rechallenged with [^177^Lu]Lu-DOTATATE: 1 did not undergo the dosimetric imaging for technical reasons, leading to the final analysis of 35 dosimetric datasets. The characteristics of the studied population are presented in Table 1. Most patients (86%) received 4 injections, except for 5 patients who had kidney impairment or platelet toxicity or were receiving retreatment with [^177^Lu]Lu-DOTATATE (2 cycles) (Table 2). In total, 146 lesions were evaluated (1–9 lesions per patient) in the liver (65%), lymph nodes (25%), mesentery (4%), pancreas (4%), and peritoneum (2%).

**TABLE 1.** Patient Characteristics

Parameter	Data related to dosimetric dataset
Median age at treatment start (y)	68
Sex	
Male	18 (51)
Female	17 (49)
Arterial hypertension	13 (37)
Diabetes	7 (20)
Carcinoid syndrome	9 (26)
Primary tumor site	
Small intestine	27 (77)
Pancreas	5 (14)
Rectum	3 (9)
Site of metastases at treatment start	
Lymph nodes	27 (77)
Peritoneum	7 (20)
Bone	12 (34)
Liver	31 (89)
Tumor grade	
1	6 (17)
2	27 (77)
3	1 (3)
Unknown	1 (3)
Treatment before [^177^Lu]Lu-DOTATATE	
Chemotherapy	12 (34)
Targeted therapy	14 (40)
Radiotherapy	4 (11)
Locoregional liver therapy	15 (43)
Somatostatin analogs	35 (100)
Surgery	11 (48)
Previous PRRT with [^177^Lu]Lu-DOTATATE	2 (6)
At least 1 treatment before [^177^Lu]Lu-DOTATATE	35 (100)
Markers of progression	
Clinical	14 (40)
Biologic	20 (59)
Radiologic	29 (83)

Data are number and percentage, except for age.

**TABLE 2.** Number of [^177^Lu]Lu-DOTATATE Cycles

Cycle	Data
1	0 (0)
2	2 (6)
3	3 (8)
4	30 (86)

Data are number and percentage.

On the basis of RECIST 1.1 and M3-CT imaging, 5 patients presented with progressive disease (14%), 8 patients had partial response (22%), and 22 patients had stable disease (64%). Overall survival was 57 mo (95% CI, 25.3 mo to not reached) and progression-free survival was 30.72 mo (95% CI, 23.00–39.43 mo). Clinical tolerance was excellent during treatment, with only grade 3 hypertension in 5 patients (14%). One patient had grade 1 nausea, and another had grade 2 nausea with grade 1 vomiting. Intercycle tolerance was excellent (grade 1–2 asthenia in most patients), except in 3 patients who presented with grade 3 asthenia. There was no grade 3–4 nephrotoxicity. Laboratory toxicities are listed in Table 3 and Table 4.

**TABLE 3.** Laboratory Toxicities According to Common Terminology Criteria for Adverse Events (Version 4.03) During [^177^Lu]Lu-DOTATATE Therapy

Blood count parameter	Laboratory toxicity			
	At least 1 grade 1	At least 1 grade 2	At least 1 grade 3	At least 1 grade 4
GFR (CKD-EPI formula) decrease	32 (91.4)	11 (31.4)	0 (0.0)	0
Hemoglobin decrease	14 (40.0)	0 (0.0)	0 (0.0)	0
Leukocyte count decrease	35 (100.0)	10 (28.6)	1 (2.9)	0
Lymphocyte count decrease	30 (85.7)	29 (82.9)	21 (60.0)	0
Platelet count decrease	14 (40.0)	4 (11.4)	1 (2.9)	0

GFR = glomerular filtration rate; CKD-EPI = Chronic Kidney Disease Epidemiology Collaboration.

Data are number and percentage.

**TABLE 4.** Variation in Laboratory Parameters Between First Injection and Month 3 and Then 12 Months After Last Injection

Blood count parameters	*n*	Before first injection	Month 3 after last injection	*P*	*n*	Before first injection	Month 12 after last injection	*P*
GFR (mL/min/1.73 m^2^)	31	72.1 (15)	72.5 (17.3)	0.921	23	70.4 (13.9)	76 (18.1)	0.247
Hemoglobin (g/dL)	32	13.4 (1.3)	12.4 (1.4)	0.009*	24	13.2 (1.2)	12.5 (1.7)	0.095
Leukocytes (×10^9^/L)	32	6.5 (2.2)	4.1 (1.2)	<0.001*	23	5.9 (1.9)	4.3 (1.2)	0.001*
Lymphocytes (×10^9^/L)	32	1.5 (0.5)	0.8 (0.3)	<0.001*	24	1.5 (0.5)	0.9 (0.3)	<0.001*
Platelets (×10^9^/L)	31	218 (68)	162.5 (51.1)	<0.001*	24	216.1 (68)	185.5 (69.1)	0.128

* *P* < 0.05.

GFR = glomerular filtration rate.

Data are means with SD in parentheses. *P* values were determined by Student *t* test.

**FIGURE 1.**
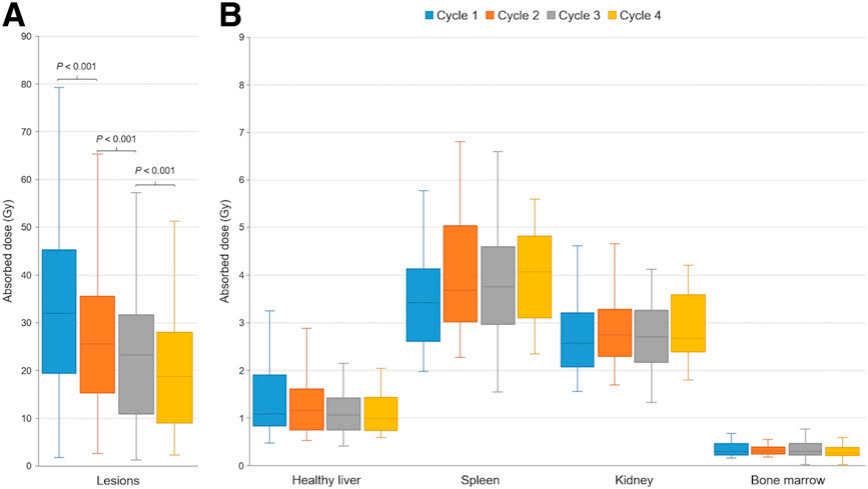
Distribution of ADs by lesions (A) and selected healthy organs (B) in 4 PPRT cycles.

**FIGURE 2.**
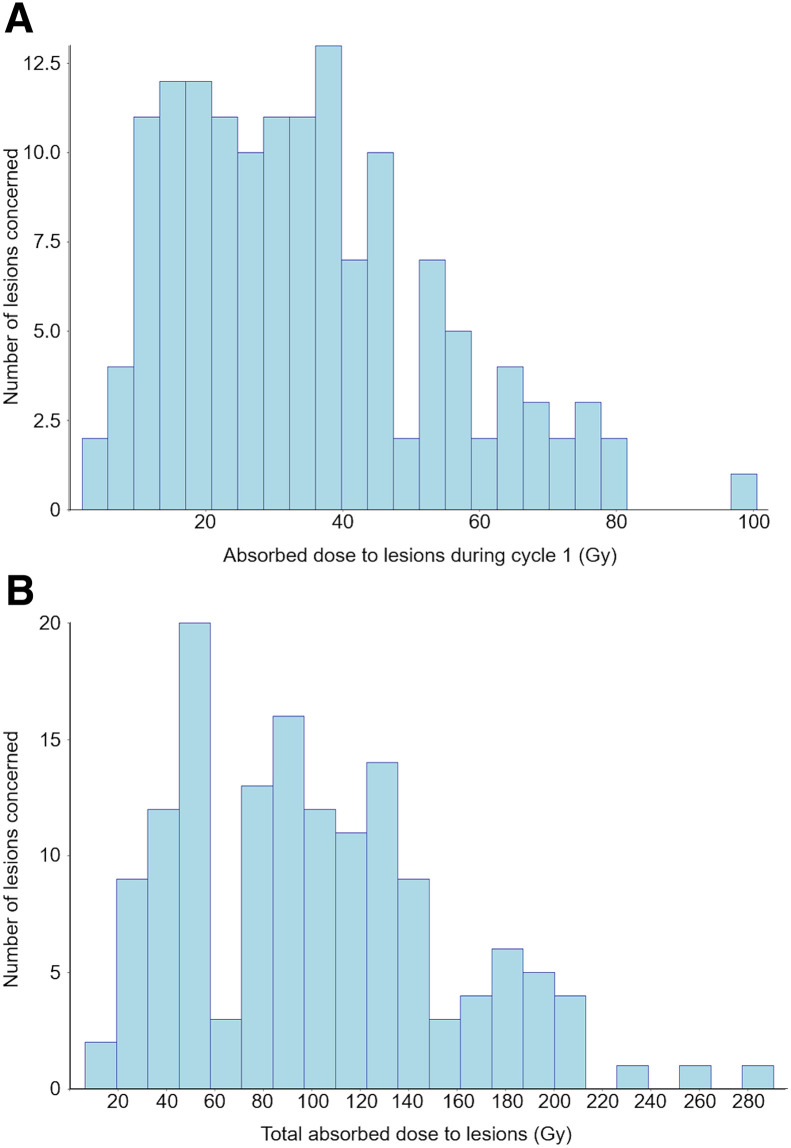
Distribution of ADs by lesions after cycle 1 (A) and after treatment end (B).

The ADs by organs and lesions are presented in Figure 1 and Table 5. The ADs by healthy organs were not significantly different among [^177^Lu]Lu-DOTATATE cycles except for the spleen (*P* < 0.05). The median ADs by the 146 lesions were 32 Gy (cycle 1), 25.6 Gy (cycle 2), 23.3 Gy (cycle 3), and 18.7 Gy (cycle 4). The ADs by lesions decreased significantly over time from cycle 1 to cycle 4 (*P* < 0.001). The median total AD by lesions during 1 full treatment course was 94.4 Gy, with a wide distribution range (Fig. 2).

**TABLE 5.** Distribution of ADs by selected Healthy Organs and Lesions Throughout Cycles and at End of Treatment Course (Total AD)

Parameter	Cycle 1 (Gy)	Cycle 2 (Gy)	Cycle 3 (Gy)	Cycle 4 (Gy)	Total AD (Gy)
Healthy liver	1.08 (0.47–9.32)	1.16 (0.52–9.34)	1.06 (0.41–5.48)	0.99 (0.59–4.19)	4.05 (2.06–27.29)
Spleen	3.42 (1.98–5.77)	3.69 (2.27–6.81)	3.76 (1.55–7.04)	4.08 (2.35–5.60)	14.28 (6.87–23.87)
Kidneys	2.73 (1.97–5.46)	2.74 (1.78–4.97)	2.74 (1.41–5.32)	2.77 (1.85–5.44)	10.77 (4.99–21.12)
Bone marrow	0.29 (0.15–1.4)	0.31 (0.18–1.06	0.29 (0.15–1.18)	0.26 (0.18–0.65)	1.07 (0.63–3.74)
Lesions	31.99 (1.77–98.49)	25.64 (2.59–73.03)	23.3 (1.22–81.73)	18.74 (2.33–74.17)	94.43 (8.73–287.89)

Values are median and range in parentheses.

### Absorbed Dose–Effect Relationship

**FIGURE 3.**
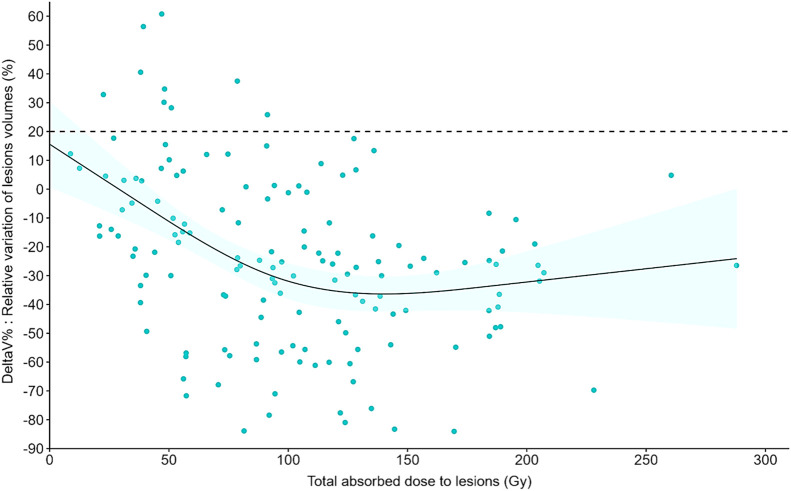
Relative tumor volume variation as function of total AD by lesions (*n* = 146). Solid line is prediction of model with 95% CI (blue haze region). Horizontal dotted line is 20% threshold. DeltaV% = variation of lesion volume.

**FIGURE 4.**
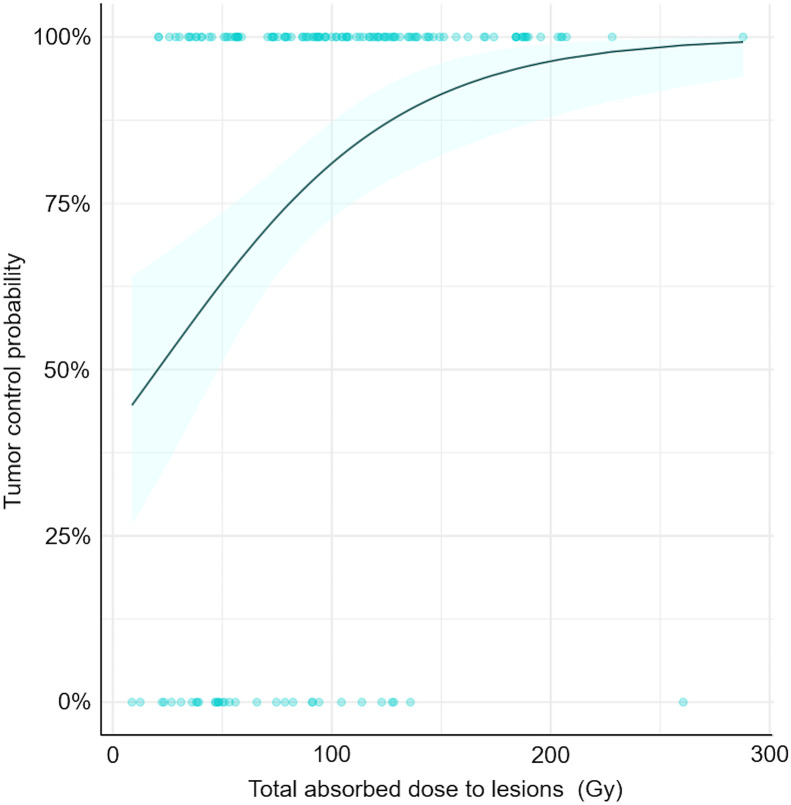
Tumor control probability. Variation of lesion volume ≤ 0% between BL-CT and M3-CT is considered as controlled tumor. Tumor control probability curve was produced using binary logistic regression model and its 95% CI.

There was a significant (*P* < 0.001) and negative correlation between the total AD by lesions and the lesion volume variations between BL-CT and M3-CT (Fig. 3). A total AD by lesions in the range of 55.8–130.7 Gy led to a decrease of 21.9% of the lesion volume. With a total AD of more than 95 Gy, all lesions were considered stable (no volume increase of >20%). The tumor control probability is presented in Figure 4.

**FIGURE 5.**
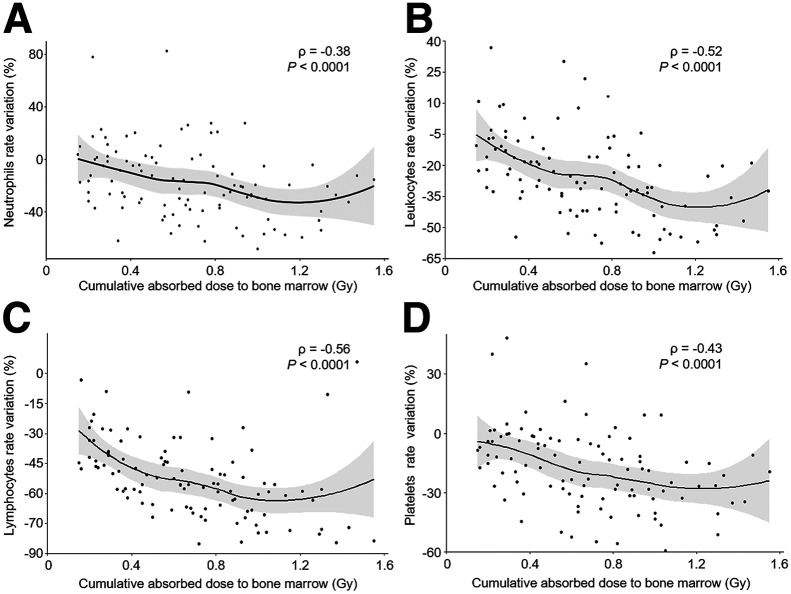
Correlation between cumulative AD by bone marrow and variations of neutrophils (A), leukocytes (B), lymphocytes (C), and platelets (D) relative to baseline. Curves are estimated using locally estimated scatterplot smoothing method, with 95% CI (gray region). Spearman correlation coefficient (ρ) is nonparametric measurement that estimates monotonic (not necessarily linear) relationship between 2 variables.

There was no significant correlation between the glomerular filtration rates and the ADs by the kidneys during treatment (Supplemental Fig. 3). The ADs by the bone marrow (Fig. 5) and the spleen (Supplemental Fig. 4) were negatively and significantly (*P* < 0.0001) correlated with variations, compared with baseline values, of neutrophil, lymphocyte, leukocyte, and platelet counts. The AD by bone marrow better correlated with a decrease of leukocytes and platelets than did the AD by the spleen. For instance, a CumAD increase by bone marrow from 0.3 to 0.9 Gy was related to a significant decrease (*P* < 0.05) of leukocytes by 14.6% (95% CI, −20.2% to −9.0%), neutrophils by 14% (95% CI, −22.0% to −6.2%), lymphocytes by 18% (95% CI, −22.3% to −13.7), and platelets by 22% (95% CI, −30.8% to −13.3%) compared with baseline values. A change in CumAD by the spleen from 4.6 to 14.0 Gy resulted in a significant decrease in platelets by 15.7% (95% CI, −22.7% to −8.6%; *P* < 0.05) and lymphocytes by 20% (95% CI, −24.6% to −16.8%; *P* < 0.05).

**FIGURE 6.**
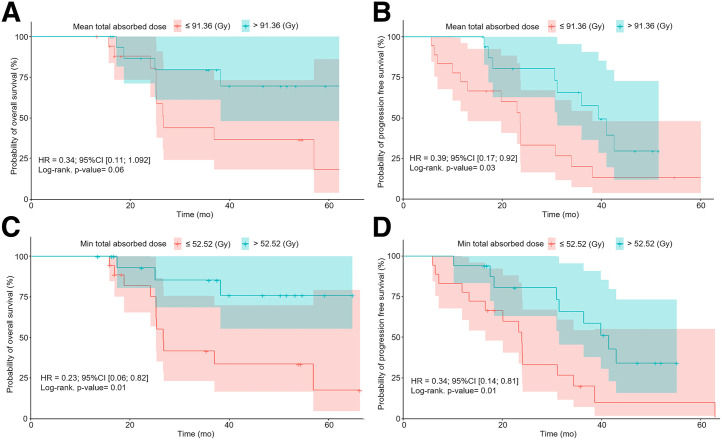
Kaplan–Meier estimates of overall survival (A) and progression-free survival (B) are shown as function of mean total AD by all lesions in each patient’s course of treatment (*n* = 35). Kaplan–Meier estimates of overall survival (C) and progression-free survival (D) are shown as function of lowest total AD in each patient’s course of treatment (*n* = 35). Min = minimum.

The dosimetric indices for lesions in patients are presented in Table 6. Patients with a mean total AD of more than 91.36 Gy by all target lesions presented a higher probability of progression-free survival (hazard ratio [HR], 0.39; 95% CI, 0.17–0.92; *P* = 0.03) but not overall survival (HR, 0.34; 95% CI, 0.11–1.09; *P* = 0.06) (Figs. 6A and 6B). The median progression-free survival was 39.4 mo (range, 31.1 mo to not reached) in patients with a mean total AD of more than 91.36 Gy and 23.6 mo (range, 13–38.2 mo) in patients with a mean total AD of less than 91.36 Gy. Patients with a Min total AD of more than 52.52 Gy by lesions had a higher probability of progression-free survival (HR, 0.34; 95% CI, 0.14–0.81; *P* = 0.01) and overall survival (HR, 0.23; 95% CI, 0.06–0.82; *P* = 0.01) (Figs. 6C and 6D). Progression-free survival and overall survival were 41 mo (range, 31.1 mo to not reached) and not reached, respectively, in patients with a minimum total AD of more than 52.52 Gy, and progression-free survival and overall survival were 23.6 mo (range, 16.3 mo to not reached) and 26.6 mo (range, 25.2 mo to not reached), respectively, in patients with a minimum total AD of less than 52.52 Gy. The Max total AD was not associated with progression-free survival and overall survival.

**TABLE 6.** Dosimetric Indices (Gy) in Patients (*n* = 35)

Mean total AD	Min total AD	Max total AD
91.36 (20.88–205.19)	52.52 (8.73–184.31)	118.59 (20.88–287.89)

Mean total AD = mean of total AD of all lesions in 1 patient; min total AD = lowest total AD value among all lesions in 1 patient; max total AD = highest total AD value among all lesions in 1 patient.

Values are median and range in parentheses.

## DISCUSSION

Here, we found a significant correlation between the AD by bone marrow and the decrease of hematologic parameters, which agreed with results from previous studies (11*,*^12^). If the baseline hematologic parameters and the stability of the AD by bone marrow over the cycles are considered, this relationship may help to better select patients and anticipate toxicities. However, one should consider that the nadir of the hematologic parameters might occur beyond day 15 after injection. The AD by the spleen was significantly higher starting from cycle 2 than it was starting from cycle 1, with a significant inverse correlation between the CumAD by the spleen and variations of hematologic parameters. Similarly, in a previous study (13), the CumAD by the spleen (15 vs. 14.3 Gy in our study) was inversely correlated with hemoglobin and platelet variations. In our sample, the CumAD by the kidneys never reached 23 Gy, which was considered an endpoint in recent PRRT trials (19*,*^20^) and was considered for external-beam radiotherapy. Moreover, patients never presented any significant glomerular filtration rate decrease during treatment and for up to 12 mo after treatment end, confirming that the kidney is not a limiting organ in PRRT with [^177^Lu]Lu-DOTATATE when delivered as 4 cycles at 7.4 GBq and amino-acid infusion.

As shown by other works, the AD by tumors was heterogeneous (from 8.73 to 287.89 Gy) (10). However, the AD to target lesions was significantly correlated with variations of their volume (*P* = 0.01). The probability of tumor control also was higher above a specific threshold: all lesions were considered as responding when they received a dose of more than 95 Gy. These results, in favor of an AD–response relationship, are in agreement with 2 retrospective studies on 24 patients with pancreatic NETs (14) and 23 patients with small intestine NETs (15), in which dosimetric results were calculated using multiple-time-point SPECT imaging. We confirmed that the AD to tumors decreased significantly over cycles, probably due to a decrease in SSTR density (15*,*^16^*,*^21^). However, we also noticed that above a certain threshold, the AD–response relationship appeared limited, with the appearance of a plateau; adding more radiation to the lesions did not seem to improve the efficacy. This may be explained by methodologic issues or radiobiologic mechanisms of resistance to ionizing radiation that require further translational studies.

Lesion dosimetric indices had a prognostic value: patients with a mean total AD by target lesions of more than 91.36 Gy were more likely to have a longer progression-free survival (HR, 0.39; 95% CI, 0.17–0.92; *P* = 0.03). Moreover, patients with at least 1 target lesion receiving a total AD of less than 52.52 Gy had significantly lower overall survival and progression-free survival. Other prognostic factors had already been identified for patients with NETs, including tumor grade (and percentage of Ki-67–positive tumor cells), tumor heterogeneity (entropy) (22), tumor glucose metabolism assessed by [^18^F]FDG PET/CT (23), injected activity (24), the size of the largest lesion (for patients receiving salvage PRRT) (25), tumor perfusion and SSTR density (26), molecular profiling (27*,*^28^), and inflammation-based indices (29). A recent retrospective cohort study with a population quite similar to ours (progressive disease, 7% vs. 14% in our study; stable disease, 70% vs. 64% in our study; partial response, 24% vs. 22% in our study) did not find that the tumor AD was predictive of patient overall survival (16). However, Alipour et al. used a different dose calculation methodology (imaging only at 24 h after infusion vs. SPECT/CT performed at different time points in our series for cycles 1 and 2 at least; no use of the lesion minimal AD index), with 68% of patients receiving at least 1 [^177^Lu]Lu-DOTATATE cycle with radiosensitizing chemotherapy, and their radiologic endpoint was not based on CT imaging but on molecular imaging of the SSTR volume. This volume reflects the tumor volume and is also correlated with SSTR expression level. This may also explain why no correlation was found between the AD by lesions and the change in molecular imaging of the SSTR volume in the previous study (16). In a recent publication, a prospective study of 37 patients with GEP-NETs who were treated with [^177^Lu]Lu-DOTATATE showed a significant increase of progression-free survival for patients whose target lesions received an AD of more than 35 Gy after cycle 1 (17). Further analysis is needed to compare results on the same basis (cycle 1), but at first sight, the presented results seem to be in the same range as ours.

Our study has some limitations, particularly its monocentric and retrospective design. It also has some methodologic limitations: as proper delineation of the lesion on noncontrast low-dose CT of SPECT/CT is not feasible in an accurate way, a constant volume was used over cycles for lesion dosimetry. That methodology may underestimate the total AD by the lesion, especially for highly responding lesions, and could, in part, explain the aspect of the plateau in Figure 3. Whether a multiple-time-point dosimetry should be performed after several administration cycles (2 in our study) is a debated question. For obvious organizational reasons, decreasing the number of cycles with full dosimetry or decreasing the number of time points per cycle would simplify the process. We are willing to consider such simplified approaches in the future and assess how they affect the correlation between the AD and the clinical outcome. Still, our results may influence the clinical management (efficacy and toxicities) of patients treated with [^177^Lu]Lu-DOTATATE for GEP-NETs. Indeed, as the AD by tumors decreases over cycles, the total AD after 4 cycles can be estimated, and these values can be compared with the patient dosimetric indices that are prognostic factors of survival. New PRRT algorithms may be proposed to deliver more irradiation to lesions (through higher injected activities per cycle or by adding more cycles) for patients with low lesion ADs after the first cycles. Moreover, as the ADs by the spleen and bone marrow are significantly correlated with variations of hematologic parameters, they could be considered as surrogate markers of toxicity during personalized treatment mainly driven by lesion dosimetry.

## CONCLUSION

The results of this study suggest that personalized dosimetry of tumors and healthy organs during treatment with [^177^Lu]Lu-DOTATATE may improve clinical outcomes and influence patient management. These results need to be validated in prospective clinical trials.

## DISCLOSURE

Emmanuel Deshayes and Kévin Hebert received fees from AAA/Novartis. Manuel Bardiès and Lore Santoro cosupervised a student sponsored by DOSIsoft. This study was supported by SIRIC Montpellier Cancer Grant INCa-DGOS-Inserm 6045. No other potential conflict of interest relevant to this article was reported.

## Notes

KEY POINTS **QUESTION:** Can tumor ADs predict outcome in patients with NETs who are treated with [^177^Lu]Lu-DOTATATE? **PERTINENT FINDINGS:** In a retrospective cohort of patients with NETs (mostly grade 2 small intestine NETs) treated with [^177^Lu]Lu-DOTATATE, tumor and healthy organ dosimetry indices are predictive of survival and correlate with toxicities. **IMPLICATIONS FOR PATIENT CARE:** These data are in favor of dosimetry implementation to improve patient selection for treatment with [^177^Lu]Lu-DOTATATE.
